# Remarks, Gleanings, and Extracts

**Published:** 1874-05

**Authors:** A. R. Kilpatrick

**Affiliations:** Texas


### REMARKS, GLEANINGS AND EXTRACTS

BY A. R. KILPATRICK, M.D., OF TEXAS.

*Fractures of Lower Extremities Treated Without Splints.*—Dr. J. F. Atkinson, of Lexington, Mo., in an article in the *Medical and Surgical Reporter*, April 18, 1874, contends that splints are not only useless, but in some cases injurious. He uses sand bags, cold water locally, and in the last stages, pasteboard strips.

[We have been of his opinion for several years, and feel quite satisfied that splints, boxes, etc., are applied too soon in all cases where they are used before deposition of provisional callurs, which is generally near the ninth day. The laity however are hard to satisfy and convince on this point, as they think every minute of delay jeopardises the limb and life of the patient. A general practitioner, where competition is active, would lose practice by attempting to conduct a case on Dr. A's plan.]

*Nux Vomica in Obstetrics.*—Dr. S. P. Sackett, Ithica, New York, in *Medical and Surgical Reporter*, strongly advocates the use of nux vomica in parturition, in place of morphine, chloroform, chloral, ergot and quinine in any case where these medicines may be indicated. Give three grains of the powder every half hour, and usually three doses will be all that is required. He avers that it alleviates suffering, regulates the pains and facilitates the delivery, obviating the use of forceps. Where the pains are irregular, he gives at once six or eight grains. Equivalent doses of any other form of the medicine can be substituted, where the powder can not be obtained.

*Poisoning by Rhus Toxicodendron.*—Many cases of this have been reported within the last year, and different plans of treatment, successful and unsuccessful, have been detailed. Acetate of lead lotion, and such remedies as usually relieve eruptions, have either no efficacy, or are injurious. Alkaline washes have a better effect. Some are relieved by bathing the parts with tincture of lobelia seeds. Dr. J. S. Stone, of London county, Va., reports two cases, in which he used a lotion made of carbolic acid, ʒiij; glicerine, ℥ij; water q. s. to make oj, mixture which was applied and gave almost instant relief and in three days they were well.

*Guarana for the Cure of Sick Headache.*—T. P. Caillouet, of Lockport, La., speaks in very high terms, in the *Phila. Med. News*, January, 1874, of guarana in powder, in doses of ten or fifteen grains, for the complete relief of sick headache. Give a dose every two hours during an attack, and take it as soon as any symptoms come on, as a prophylactic. It relieves many instantly and some have no return for months and years after having used the medicine

*The Betel Nut an Anthelmintic.*—The inhabitants of Bombay, grate the betel nut, as we do nutmegs, and give a teaspoonful of the powder, floating on milk, or mingled in clarified butter. It is given before breakfast, after the patient has fasted twelve hours, and it acts without any adjuvant, bringing away lumbrici and tape-worms It acts on dogs in the same doses.—*Medical and Surgical Reporter*.

*Biliary Calculi Dissolved by Chloroform.*—Dr. E. Burd, of Lisbon, Iowa, recommends chloroform in treating cases of impacted biliary calculi, giving fifteen to thirty drops every half hour. He also uses Venice turpentine to one of ether, as a solvent during the interval between paroxysms of the disease.—*Med. and Surg. Reworter*

*Ablation of Mammæ Causes Sterility.*—A French physician, or scientist removed the mammary glands from several Indian pigs, without any serious injury to the animals, except that they remained sterile.

*Hypodermic Injection of Ergotine in Uterine Fibroid.*—Professor Hildebrandt, of Königsburg, Germany, states that while using this remedy for metrorrhagia, he discovered it also reduced fibroid tumors which were co-existent. Dr. Beugelsdorf repeated the experiments with similar results, and reports four cases of uterine fibroma which he treated. They both used three parts of watery extract of secale cornutum to seven parts of glycerine and water. The advice is to insert the canula into the muscles and diffusing the fluid by manipulation, so as to prevent phligneous inflammation.

Two years ago I treated a negress aged fifty, large and fleshy, who was suffering with uterine pain and high fever. On digital examination, at the first visit, found the uterus enlarged to the size of a new-born child's head, the os dilated and something protruding from it, with some hemorrhage present. She had been sick some weeks, very much prostrated, and I made no spceular examination, as I thought she would soon die. I used astringent vagical enemata, and gave large doses of tinct. ergot with acet. plumbi, kino and spts. lavender comp. She improved rapidly, and, contrary to expectations, she soon got up. Some few weeks after that she sent for me again, to treat her for remittent fever, and I then supposed that the uterus was involved, and, on exination, discovered that the organ was of normal size and in position, and the tumor I had felt (more than once before) had disappeared. In a few weeks more she moved to another county and I lost sight of her.

*Tic-douloureux Cured by Ice.*—Dr. B. M. Walker, of Plymouth, N. C., reports, in the April number of *American Journal of Medical Sciences*, a case of a lady, aged sixty, who had been suffering at intervals for over five years, and under treatment, using various remedies and attending mineral springs with only partial and temporary relief. Dr. W. saw where Dr. Winternitz treated a case successfully by the local application of ice. The patient applied the ice directly to the seat of pain, which was in the 5th pair of nerves, at the same time holding brandy in her mouth. Although the pain was regularly paroxysmal and intermittent, it never returned. The ice was kept to the part as long as it could be borne.

*Treatment of Chloroform Asphyxiation.*—Professor Nelaton, some years ago, relieved a case of this kind, by placing the patient head down and feet up, so as to fill the cerebral congestion. More recently the practice has been adopted and recommended by Doctor Campbell, of Paris. He considers that death arises through syncope due to cerebral anæmia. This plan also tends to drive from the lungs and trachea pent up vapors of chloroform and mucus which were increasing the asphyxia.—*Lancet.*

